# Chronic Myeloid Leukemia and Cesarean Section: The Anesthesiologist's Point of View

**DOI:** 10.1155/2018/3138718

**Published:** 2018-09-18

**Authors:** Houssam Rebahi, Mourad Ait Sliman, Ahmed-Rhassane El Adib

**Affiliations:** Department of Anesthesia, Intensive Care Medicine, Emergency & Simulation, Medical School of Marrakech (Cadi-Ayyad University), Mother & Child Hospital, Mohammed VI Teaching Hospital, Marrakech, Morocco

## Abstract

**Background:**

Chronic Myeloid Leukemia (CML) is a myeloproliferative neoplasm related to chromosomal reciprocal translocation t(9;22). Tyrosine kinase inhibitors (TKIs) such as imatinib have drastically revolutionized the course and the prognosis of this hematologic malignancy. As we know, the association pregnancy-CML is an infrequent situation. Also the use of TKI in pregnant women is unsafe with a lack of alternatives and effective therapeutic options. Thus its cessation during gestation puts those patients at high risk of developing blast crisis characterized by poor outcomes.

**Case Report:**

A 37-year-old pregnant woman, gravida 2, para 2, with a previous cesarean section in 2011, presented to the obstetric unit. Her medical past revealed that she is a newly diagnosed patient with CML managed by TKI during her preconception period. Due to the perilous use of TKI during her pregnancy, a switch to interferon-*α* administration was adopted. But after the completion of 36 weeks of gestation, disease progression (relapse with blast crisis), attested by biological worsening, a white blood cell count = 245000/mm^3^ with 32% blasts in the peripheral blood, urged the medical team to opt for cesarean delivery. She underwent general endotracheal anesthesia without any perioperative incidents and gave birth to a healthy newborn. Ten days later, the patient was started on TKI.

**Discussion:**

Although data on this specific and challenging situation are limited, this case highlights the difficulties encountered by the anesthesiologists when choosing the accurate anesthetic strategy and how important it is to weigh the risks and benefits inherent to each technique. Above all, taking into consideration the possible central nervous system (CNS) contamination by circulating blast cells when performing spinal or epidural approach is primordial. This potential adverse event (CNS blast crisis) is extremely scarce but it is responsible for high rates of morbidity and mortality.

## 1. Introduction

CML management during pregnancy remains controversial. TKIs are an effective first- or second-line therapy and have demonstrated increased overall survival [1, 2]. However, continuing this molecular targeted therapy during pregnancy, particularly throughout the first trimester, seems to be unsafe and harmful for the fetus, as it belongs to US Food and Drug Administration (FDA) Pregnancy Category D given this potential embryo-fetotoxicity [1, 3].

These safety concerns and the absence of clear and valid recommendations compel most of medical teams to cease TKIs therapy during pregnancy [4–6], which exposes these patients to inevitable disease progression, possibly towards a blastic phase which is life-threatening and difficult to treat [1, 7].

The following case describes the challenges encountered by the hematologist, obstetrician, and anesthesiologist faced with a pregnant woman with CML.

## 2. Case

Six years after an uncomplicated cesarean delivery of a first child and one year after being diagnosed with CML, a 37-year-old woman presented to our institution at 36 weeks of gestation with worsening fatigue associated with abdominal discomfort.

The couple had rejected the option for medical-assisted abortion during the first trimester and, due to her low risk of CML progression determined by an European Treatment and Outcome Study (EUTOS) score of 86 [7], her hematologist opted for imatinib cessation, which was given initially at a dose of 400 mg per day, during the first and second trimester. She received interferon-*α* in the later half of pregnancy. Response to treatment was assessed regularly and at the end of 35 weeks she was found to have a palpable spleen (increased from 4 cm to 8 cm below the lower left costal margin) and leukocytosis at 245,000WBC/mm3 including 32% blast cells. Platelet count and hemoglobin concentration were within normal ranges. Bone marrow aspiration was performed and the patient was diagnosed with CML in acute phase. Cytogenetic analysis of the bone marrow cells by using the Giemsa Banding technique revealed the karyotype 46,XX,t(9,22)(q34;q11.2) in 100% of the analyzed cells without any additional abnormality. FISH analysis was not performed.

Due to the significant deterioration and urgent need for chemotherapy initiation, cesarean delivery was planned for the end of 36 weeks, which was uneventful under general anesthesia. The rate of circulating blasts made the choice of general anesthesia mandatory and judicious rather than a risky perimedullary anesthetic technique. The newborn was healthy and did not require any medical interventions. Postoperatively, she received a multimodal analgesia and an effective thromboprophylaxis. Afterwards, she was started on treatment with imatinib, 800 mg daily, without any satisfactory response. Because of disease progression, the patient received hydroxyurea as palliative treatment with partial response.

## 3. Comments

It is well known that CML is a myeloproliferative neoplasm associated with the specific cytogenetic hallmark of the Philadelphia chromosome (reciprocal translocation t(9;22)(q34;q11.2)), found in almost 95% of patients [10–12]. Untreated, this disorder displays an inevitable propensity to evolve from a chronic into an accelerated and ultimately a blastic phase. Undoubtedly, blast crisis, defined as >30% myeloblasts in the circulation [10–12], represents a critical turning point in the CML course triggering a rapid clinicobiological worsening with poor outcomes [10].

Up to this point, the gold standard strategy to slow down disease progression and improve life expectancy of patients diagnosed with CML was a life-long TKI regimen. In fact, it may be hazardous to interrupt the imatinib course because of the significant risk of relapse [10, 11]. Although allogenic stem cell transplantation remains the only curative treatment, it should be reserved for patients not achieving an optimal and sustained response to TKIs due to its frequent and severe procedural-related complications [10, 11].

Given that CML within the setting of pregnancy is a relatively rare situation (approximately 1/75000 pregnancies), appropriate approaches are usually best decided on a case-by-case basis rather than strict protocols consistent with the evidence [10–12]. In fact, the decision-making process in such cases is a current topic of debate and is characterized by lack of uniformity and standardization [10].

Consequently, experts recommend that women with CML adhere to an effective contraceptive plan. However in the case of planned pregnancy or rejection of medically assisted abortion, a medical team should involve every care provider, including the pregnant woman, to fully explore the risks and benefits of the treatment options and choose the best plan for both the mother and fetus [3, 4, 11, 13]. The embryo-fetotoxic effects of TKIs, observed in 12 infants among 180 reported pregnancies in one dedicated study, are still debated and, thus, some authors propose to continue imatinib during pregnancy in an effort to avoid the high risk of both cytogenetic and clinical relapses [5, 14, 15]. Others recommend TKI discontinuation throughout the first trimester. Various other therapies including hydroxyurea, interferon, various combinations of chemotherapeutic agents, and intensive leukapheresis have been suggested to lower the risk of progression, induce rapid disease control, or possibly reverse the blastic stage for those who are unable or unwilling to continue with TKI therapy, but these have not been validated [16, 17].

Additionally, this case depicts the challenges faced by anesthesiologists with a patient in blast crisis who is scheduled for cesarean section [11, 18]. It remains unclear if blast crisis is a contraindication to spinal anesthesia, so obstetric anesthesiologist should be aware of some key findings that may dictate the choice of safe anesthetic technique. Many authors compare this situation with traumatic lumbar punctures performed at the start of chemotherapy in young patients with acute lymphoblastic leukemia (ALL) which were linked to significant increased CNS relapse and decreased event-free survival. This is likely due to the introduction of blood to cerebrospinal fluid (CSF) as the number of myeloblasts found in CSF has been correlated with a worse prognosis [19–21]. A tissue coring phenomenon may also be responsible for spreading cancer cells from extramedullary blast infiltrates (skin, ligaments, or muscles) to the epidural or dural spaces when advancing the needle (Table 1) [8, 9, 18, 20, 21]. This last issue persists whether or not an introducer is used [22]. Hence, many authors believe that, in the presence of ≥5% of circulating blasts, general anesthesia is the safer choice for cesarean delivery [11, 18].

## 4. Conclusion

The management of a safe pregnancy in women diagnosed with CML includes many significant challenges. The termination of TKI therapy in the first trimester, owing to its potential teratogenicity, puts these patients at high risk for developing a blast crisis, a serious and often life-threatening event that requires prompt and aggressive management. Additionally, a continuous risk assessment for both mother and fetus is mandatory, and response to a chosen approach should be regularly monitored and recorded. Finally, it appears desirable to avoid spinal anesthesia when the intravascular blast count exceeds 5% to maximize patient safety and improve outcomes.

## Figures and Tables

**Table 1:** Tissue coring incidence depending on type of spinal needle.

Campbell [8]	Quincke 25G: 80%	Whitacre 25G: 41%	
Puolakka [9]	Quincke 27G: 56%	Whitacre: 37%	Sprotte: 37%
